# Rapamycin alleviates inflammation and muscle weakness, while altering the Treg/Th17 balance in a rat model of myasthenia gravis

**DOI:** 10.1042/BSR20170767

**Published:** 2017-07-17

**Authors:** Feng Jing, Fei Yang, Fang Cui, Zhaohui Chen, Li Ling, Xusheng Huang

**Affiliations:** 1 Department of Neurology, Chinese PLA General Hospital, Beijing 100853, China; 2 Department of Neurology, The 309th Hospital of Chinese PLA, Beijing 100091, China

**Keywords:** Autoimmune disease, animal models, myasthenia gravis, rapamycin

## Abstract

Myasthenia gravis (MG) is an autoimmune disease commonly treated with immunosuppressants. We evaluated the novel immunosuppressant, rapamycin (RAPA), in a rat model of experimental autoimmune MG (EAMG). Mortality rates in the RAPA (12%) were significantly down compared with the EAMG (88%) or cyclophosphamide (CTX) (68%) intervention groups. Muscular weakness decreased after both RAPA and CTX treatment. However, Lennon scores were lower (1.74 ± 0.49, 3.39 ± 0.21, and 3.81 ± 0.22 in RAPA, CTX, and EAMG groups, respectively), and body weights (203.12 ± 4.13 g, 179.23 ± 2.13 g, and 180.13 ± 5.13 g in RAPA, CTX, and EAMG groups, respectively) were significantly higher, only in the RAPA group. The proportion of regulatory T cells (Treg) significantly increased, while that of Th17 cells significantly decreased in the RAPA group compared with the EAMG group. In comparison, CTX intervention resulted in increased Th17 but significantly decreased Tregs. Hence, RAPA can be more effectively used in comparison with CTX to treat MG, with an efficacy higher than that of CTX. In addition, our results suggest RAPA’s efficacy in alleviating symptoms of MG stems from its ability to correct the Treg/Th17 imbalance observed in MG.

## Introduction

Myasthenia gravis (MG) is a T-cell-dependent and B-cell-mediated autoimmune disease, which is pathologically characterized by a neuromuscular transmission disorder resulting from damage in acetylcholine receptors (AChRs) on the post-synaptic membrane [1]. CD4^+^ T cells and cytokines play critical roles in the pathogenesis of MG [2]. Native CD4^+^ T cells can be divided into four subsets: Th1, Th2, Th17, and regulatory T (Treg) cells. Treg cells play an important role in maintaining immune tolerance and balance [3]. Reduction in the number of Treg cells may increase the risk of autoimmune diseases, and studies indicated that the number of Treg cells is reduced in MG pathogenesis [4,5]. Th17 cells play a pivotal role in mediating inflammatory reactions and promoting autoimmune reactions [6]. Patients with MG have been shown to have an increased population of Th17 cells [7]. Cumulatively, this indicates that a Treg/Th17 imbalance (low Treg and high Th17 T-cell subsets) may be involved in the pathogenesis of MG.

Rapamycin (RAPA) is a macrolide immunosuppressant, which is widely used in the treatment of organ rejection after transplantation, cancer, and autoimmune diseases, with confirmed safety and efficacy. The binding site of RAPA is the mammalian target of RAPA (mTOR). Activation of mTOR protein initiates the downstream signaling pathways, which is a critical process for the differentiation of CD4^+^ T-cell subsets. Thus, inhibition of the mTOR signaling pathway in turn inhibits the production of Th17 cells [8] and induces the proliferation of Treg cells [9]. mTOR pathway inhibitors can improve the symptoms of a variety of autoimmune diseases [10-12]. Since MG pathogenesis seem to be driven by high Th17 and low Treg, it can be envisaged that RAPA will be an optimal therapeutic option to treat MG, given RAPA’s function in boosting Treg population and suppressing Th17 differentiation [8,9].

However, no study has yet evaluated the use of mTOR inhibitors, RAPA included, for the treatment of MG. This, in the present study, for the first time, we attempted to use RAPA to treat experimental autoimmune MG (EAMG) and perform a systematic evaluation of treatment effectiveness. We analyzed the dynamics of T-cell subsets before and after treatment with RAPA and preliminarily studied the possible therapeutic mechanisms in order to explore new approaches for the treatment of MG.

## Materials and methods

### Animals

Given that sex (more prominent in females compared with males) [13], age (6–8 weeks old rats are most susceptible, with older rats being progressively resistant to disease induction) [13,14], and strain (Lewis strain represents susceptible strains to autoimmune responses, but develop milder, non-fatal disease and hence is the preferred strain for EAMG) [15] are the most prominent contributing factors to the severity of EAMG [16]. Specific pathogen-free, inbred, 6–8-week old, female Lewis rats, with an average weight of 159.36 ± 8.79 g, were used in the experiments. The animals were purchased from Beijing Vital River Laboratories (Beijing, China; license number: SCXK (Beijing) 2012-0001). The animal husbandry and operations were performed in the Experimental Animal Center at Chinese People’s Liberation Army (PLA) General Hospital, with the animals being housed in a floor area of ≥800 cm^2^ and a height of >17.5 cm, with three animals per cage (in accordance with guidelines of the National Research Council (U.S.A.) Committee for the Update of the Guide for the Care and Use of Laboratory Animals) [17]. All experiments were approved by the Institutional Animal Care and Use Committee of PLA General Hospital and were conducted following its guidelines.

### Reagents and instruments

The rat-derived 97–116 peptide chain of the AChRα subunit (D G D F A I V K F T K V L L D Y T G HI) was synthesized by Nanjing GenScript Technology Co. Ltd. (Nanjing, China), with a molecular weight of 2252.57 Da and a purity of >95%. Complete (CFA) and incomplete (IFA) Freund’s adjuvant were purchased from Sigma–Aldrich (St. Louis, MO, U.S.A.). *Mycobacterium tuberculosis* H37RA powder, AChR-Ab ELISA test kit (rat), PBS buffer, and RAPA were purchased from DIFCO (Franklin Lakes, NJ, U.S.A.), Hanke-Hengyu Biotechnology Limited (Beijing, China), Jin-Shan Golden Bridge Biotechnology Co. (Beijing, China), and LC Laboratories (Woburn, MA, U.S.A.), respectively. Rat CD4^−^ FITC, rat CD25-phycoerythrin (PE), rat Foxp3-allophycocyanin (APC), rat IgG1K isotype control-PE, rat IgG2a isotype control-APC, Foxp3 buffer set, red blood cell (RBC) lysing buffer, rat interleukin (IL)-17A-APC, and cell stimulation cocktail (containing protein transport inhibitors, 500×) were supplied by eBioscience, Inc. (San Diego, CA, U.S.A.). Cyclophosphamide (CTX) injections, neostigmine, atropine, and 10% chloral hydrate solution were purchased from the Pharmacy Department at the PLA General Hospital. RPMI-1640 medium was prepared in our laboratory. The instruments used in this experiment included a microplate reader (Bio-Kinetics Reader, Microplate EL 312e; BioTek Instruments, Inc.; Winooski, VT, U.S.A.), a flow cytometer (BD LSRII; BD; Franklin Lakes, NJ, U.S.A.), and an electroneurophysiology recorder (Keypoint; Frederiksberg, Denmark).

### Establishment evaluation and end point of the animal model

The rat EAMG model, an animal model of human MG, was established as described previously [18]. The advantages of using the EAMG model is that it mimics the human disease in terms of immuopathologic and clinical manifestations [19,20]. Moreover, it is widely used for determining efficacy of potential therapeutic strategies [18-20].

First, R97–116, CFA, and PBS were mixed at a ratio of 1:1.5:1.5 and shaken vigorously to prepare an emulsion solution. Next, 1 mg of H37RA dry powder was added to 200 μl of the emulsion solution. After anesthesia, a subcutaneous injection was given using a microsyringe at multiple sites in the rat footpad, back, and abdomen. Each rat in the model group was injected with 200 μl of mixed emulsion and each rat in the blank control group was injected with PBS at the same sites. Intensive immunization was carried out at days 30, 45, and 60. In the intensive immunization scheme, R97–116, IFA, and PBS were mixed in a ratio of 1:1.5:1.5 and H37RA dry powder was added at a dose of 1 mg/200 μl. The rats in the model group were subcutaneously injected with 200 μl of the prepared mixture at multiple sites in the footpad, back, and abdomen. The rats in the blank control group were injected with the same amount of PBS.

#### Clinical evaluation

Clinical evaluation was conducted based on the EAMG clinical grading scales in animal models (rat) developed by Lennon et al. [21]. The grading criteria were as follows: grade 0, no symptom of weakness; grade 1, mild reduction in movement activity, weak biting, weakened cry, or ability to grasp, with easy fatigue that is particularly pronounced after repeated grasping activities; grade 2, pronounced symptoms of weakness, presence of a tremor before the grip, the head tending to be low, humped back posture, weak grasping; grade 3, manifestation of severe muscle weakness before grasping, inability to grasp, moribund state; grade 4, death. When the symptoms were between two grades, they were graded as 0.5, 1.5, 2.5, or 3.5, accordingly.

#### Neostigmine test

For the neostigmine test, each experimental rat was administered with neostigmine (37.5 μg/kg) and atropine (15 μg/kg) by intraperitoneal injection, and changes in the symptoms of the rats were observed every 10 min for 1 h after the injection.

#### Low-frequency repetitive nerve stimulation test

The repetitive nerve stimulation (RNS) test was conducted as previously described [22].

#### Anti-AChR antibody assay

The serum antibody titer was determined using a commercially available rat AChR antibody ELISA kit. A blood sample (0.5 ml) was collected from the tail vein of the animal and serum was obtained after centrifugation. Serum samples and standards were diluted and the serum antibody titer was determined according to the manufacturer’s instructions. Briefly, the diluted solution was added to the detection plate (50 μl/well). The sample was incubated at 37°C for 1 h, followed by washing with PBS three times. Subsequently, reagent A (enzyme-labeled antibody) was added to each reaction well, and the mixture was incubated at 37°C for 30 min, followed by washing with PBS thrice. After the addition of reagent B (chromogenic reagent), the reaction was allowed to proceed at room temperature for 20 min, followed by washing with PBS three times. In each well, 50 μl of stopping solution was added to stop the reaction. The optical density of the test sample and the standard was determined with a microplate reader at a wavelength of 450 nm.

#### End point of animal models

In order to minimize the suffering in experimental animals, we killed terminally sick animals prior to the end of the experiments. We monitored and recorded the health condition of the animal models every 8 h. The animals were given a human humane end point once they were found too sick to survive. When severe muscle weakness appeared or the Lennon score was ≥ grade 3, the animals would be killed.

### Drug intervention

After confirming that the model was successfully established, EAMG rats were used for drug testing. EAMG rats were randomly divided into the RAPA group (*n*=25), the CTX group (*n*=25), and the EAMG control group (*n*=25). Normal rats of the same age were used as blank controls (*n*=25). The rats in the RAPA and CTX groups were administered 1 mg/kg per day and 30 mg/kg per day of drugs by intraperitoneal injection, respectively. The rats in the EAMG control group and blank control group were injected with saline. The drug trial period lasted for 10 days.

### Flow cytometry assay

At the end of the experiment, peripheral blood samples were collected from the four groups of rats to measure the Treg/Th17 cell ratio and the number of CD4^+^ T cells.

#### Treg detection

CD4-FITC antibody (0.125 µg) followed by CD25-PE (0.06 µg) antibody were added to 500 μl of heparinized peripheral blood. To ensure accurate results, an isotype control tube was set up. An equal amount of rat IgG1K isotype control-PE was added. The mixture was mixed by shaking and the reaction was allowed to proceed in the dark at room temperature for 20 min. Two milliliters of RBC lysing buffer was added to each tube, the sample was mixed, and the lysis reaction was allowed to proceed at room temperature for 10 min. The mixture was centrifuged at 300×***g*** for 5 min and the supernatant was removed, followed by addition of 1 ml of PBS and centrifugation. Cells were fixed by adding 500 µl of fixation/permeabilization solution (included in the Foxp3 buffer set) in the dark at 4°C for 30 min, followed by centrifugation at 300×***g*** for 5 min. Subsequently, 0.5 µg of Foxp3-APC antibody was added, while 0.5 µg of rat IgG2a isotype control-APC was added to the isotype control tube. The mixture was mixed and incubated at 4°C in the dark for 30 min. After addition of 2 ml permeabilization buffer, the sample was centrifuged at 300×***g*** for 5 min, and the supernatant was discarded. Then, 0.3 ml of PBS was added and the sample was mixed by gentle shaking. Finally, the cells were resuspended and flow cytometry was performed for detection and analysis.

#### Th17 detection

RBC lysis was performed in 500 µl of heparinized peripheral blood sample and 2 ml RPMI-1640 medium was added. After mixing and centrifugation, the supernatant was discarded. In each tube, 1 ml RPMI-1640 medium was added and the mixture was mixed and transferred to culture plates. After adding 2 μl of cell stimulation cocktail, the cells were incubated at 5% CO_2_ and 37°C for 4 h, followed by centrifugation at 300×***g*** for 5 min. The supernatant was discarded and the sample was washed once with PBS. To ensure accurate results, an isotype control tube was set up. After addition of CD4-FITC (0.125 µg) antibody, the mixture was mixed and the reaction was allowed to proceed at room temperature in the dark for 20 min, followed by washing with PBS. Then, 500 µl of fixation/permeabilization solution was added and the reaction was carried out in the dark at 4°C for 30 min. The mixture was centrifuged at 300×***g*** for 5 min and the supernatant was discarded. In each tube, 2 ml of permeabilization buffer was added, and the mixture was centrifuged at 300×***g*** for 5 min. Subsequently, 0.125 µg of IL-17A-APC antibody was added, while 0.125 µg rat IgG2a isotype control-APC was added to the isotype control tube. The mixture was incubated at 4°C in the dark for 30 min. After addition of 2 ml permeabilization buffer, the mixture was centrifuged at 300×***g*** for 5 min and the supernatant was discarded. Cells were resuspended in 0.3 ml of PBS, and flow cytometry was used for detection and analysis.

### Statistical analysis

To confirm that the experimental analysis was appropriately powered, G*Power [23,24] was used to calculate the sample size. Given error probability as 0.05 (two-tailed), power as 0.95, and effect size as 0.60, sample size per experimental group was calculated as 25. Data analysis was conducted using SPSS17.0 statistical software (SPSS, Chicago, IL, U.S.A.). The intergroup positive rates are expressed as percentages and the intergroup differences were tested by χ^2^ tests and Fisher exact tests. In addition, two-tailed *t* tests were used for comparisons between continuous variables. *P*<0.05 was considered statistically significant.

## Results

### Assessment of the EAMG animal model

**Figure 1:**
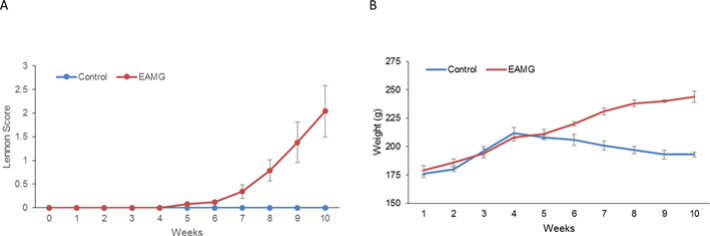
Comparison of Lennon score and weights between the EAMG (circles) and control group (squares) Compared with the control group, the EAMG rats gradually showed symptoms of weakness and weight loss after immunization with R97–116 peptide chains.

After the first inoculation, no significant change was observed between the EAMG and control groups. Ten days after the first intensive immunization, the rats in the EAMG group gradually exhibited loss of body weight, dry hair, and MG symptoms, including reduced activity, weakness in crawling and grip, weak bite and cry, and reduced food intake. Seven weeks later, the weakness symptoms of the rats in the EAMG group became more apparent, including significantly decreased activity, a humped back when standing still, and an obvious tremor. The Lennon scores of the animals in the EAMG group increased gradually (Figure 1A) and body weights declined over time (Figure 1B). Ten weeks after immunization, body weights of the EAMG and control rats were significantly different (194.29 ± 4.32 g compared with 247.54 ± 3.32 g; *P*<0.05). The difference in terms of Lennon score was also statistically significant (*P*<0.05).

The weakness symptoms of the EAMG rats improved after intraperitoneal injection of neostigmine + atropine. With regard to low-frequency RNS, 77.83% of the EAMG rats exhibited a decrease in the compound muscle action potential (CMAP) amplitude, which was not observed in the control group. All the rats in the experimental model group were positive for the AChR-Ab test, while those in the control group were all negative. These results indicated that the EAMG model rat was successfully established.

### Drug intervention

**Figure 2:**
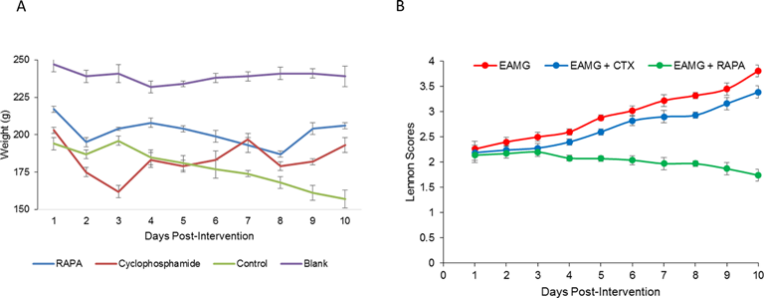
Comparison of weights and Lennon score between RAPA, CTX and EAMG group after drug intervention (**A**) Comparison of weight between the RAPA group (circles), CTX group (squares), and EAMG group (cross) after drug intervention. The weights of the animals in the RAPA and CTX groups were both higher than that of the animals in the EAMG group (*P*<0.05). (**B**) Comparison of Lennon scores between the RAPA group, CTX group, and EAMG group post-drug intervention. While Lennon scores in the RAPA and CTX groups were both lower than that in the EAMG group (*P*<0.05), suggesting the curative effect of both drugs. However, compared with the CTX group, the RAPA group showed lower Lennon scores and higher weight gain, suggesting that the therapeutic effect of RAPA was superior to that of CTX.

At the end of the intervention, the mortality rates of the RAPA, CTX, and EAMG control groups were 12.00% (3/25), 68.00% (17/25), and 88.00% (22/25), respectively, whereas no rat died in the blank control group (0/25). The mortality rate was significantly down in the RAPA group compared with either the EAMG or CTX group (*P*<0.05). The body weights of rats in the RAPA, CTX, and EAMG control groups were 203.12 ± 4.13 g, 179.23 ± 2.13 g, and 180.13 ± 5.13 g, respectively, showing a significant increased weight in the RAPA group (Figure 2A). The Lennon scores of the RAPA, CTX, and EAMG groups were 1.74 ± 0.49, 3.39 ± 0.21, and 3.81 ± 0.22, respectively. The scores of the RAPA and CTX were both significantly lower than that of the EAMG group (*P*<0.05), suggesting the curative effect (Figure 2B). We also observed significant differences (*P*<0.05) between the RAPA and CTX groups (Figure 2B), indicating a more efficacious outcome in the RAPA intervention group. Compared with the CTX group, the RAPA group showed a lower mortality rate and Lennon score as well as a higher weight gain, suggesting that the therapeutic effect of RAPA was superior to that of CTX.

### T-cell populations in peripheral blood

**Figure 3:**
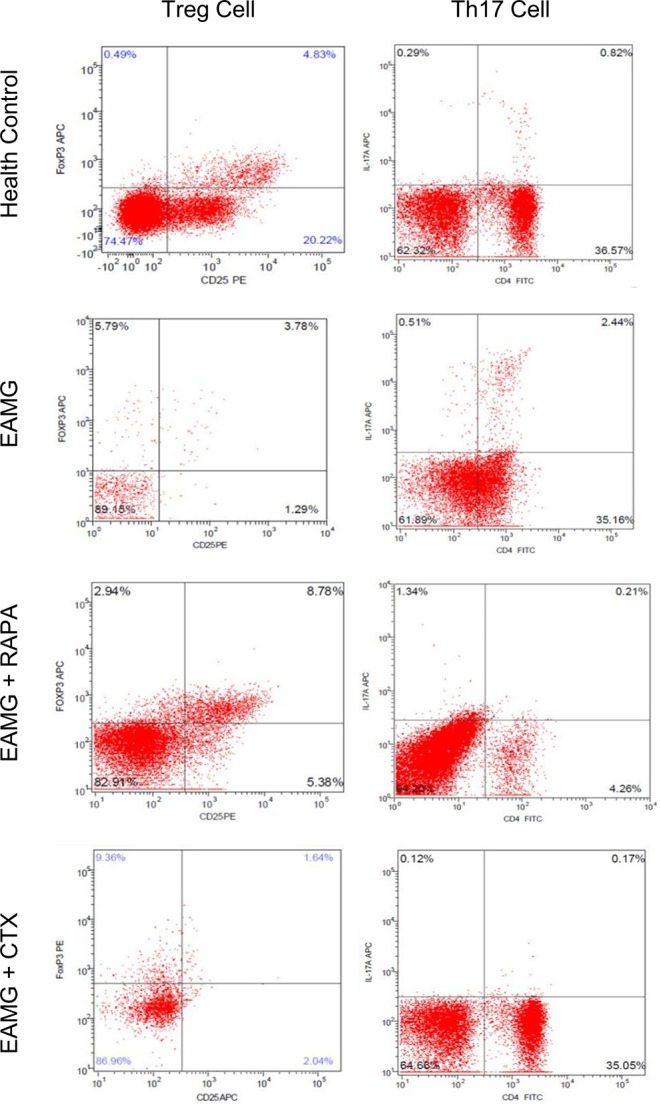
Proportion of Treg and Th17 cells measured by flow cytometry Compared with the healthy control group, the proportion of Treg cells in the EAMG group was reduced (*P*<0.05) and the proportion of Th17 cells was significantly increased (*P*<0.01), suggesting the presence of a Treg/Th17 cell imbalance in the pathogenesis of EAMG. Compared with EAMG, the proportion of Th17 cells in both the RAPA and CTX groups decreased after the intervention (*P*<0.01), which might have contributed to the symptom improvement. The proportion of Treg cells in the RAPA group increased, while that of Th17 cells decreased (*P*<0.01). Both the proportions of Treg and Th17 cells in the CTX group decreased (*P*<0.01).

We drew peripheral blood samples from the living rats in the four groups to assess the T-cell populations (RAPA, *n*=22; CTX, *n*=8; EAMG, *n*=3; blank, control *n*=25) at the end of the drug intervention on day 10. Compared with the blank control group, the EAMG control group showed a decreased proportion of Treg cells and an increased proportion of Th17 cells. After drug intervention, the Th17/CD4^+^ T-cell ratios were significantly reduced in the RAPA and CTX groups (*P*<0.01). Compared with the EAMG control group, the RAPA group exhibited an increased proportion of Treg cells, whereas the proportion of Treg cells in the CTX group was significantly reduced (Figure 3). These results cumulatively indicated that RAPA intervention was the only experimental group in which the Th17/Treg imbalance observed in MG was corrected.

## Discussion

Treg and Th17 cells are two newly discovered CD4^+^ T-cell subsets, which have been in focus in recent immunological studies. CD4^+^CD25^+^FoxP3^+^ regulatory T cells are also known as Treg cells, which mature in the thymus and mainly exist in the thymus and peripheral lymphoid organs. Treg cells play a very important role in maintaining immune balance and regulating immunological self-tolerance [25]. The thymus achieves immune tolerance to self-antigens by clearing the activated autologous T cells. However, a portion of pathogenic T cells cannot be completely cleared, which requires regulation through peripheral tolerance mechanisms. The most important tolerance mechanism is the regulation by Treg cells [26]. Defects in the number and function of Treg cells are characteristics of various autoimmune diseases, and disease progression can be reversed by increasing the number of Treg cells with normal function [27]. Increasing evidence suggests that changes in the number and function of Treg cells are involved in the pathogenesis of MG in human cases and animal models [28,29]. In the present study, we determined that the percentage of Treg cells in the peripheral blood of EAMG rats was far lower than that of normal rats, suggesting that the pathogenesis of MG might be associated with the damage of immunological self-tolerance induced by the reduced proportion of Treg cells.

Th17 cells have a strong pro-inflammatory effect and are widely involved in the occurrence and development of inflammation, cancer, organ rejection after transplantation, and autoimmune diseases. Studies revealed that levels of Th17 cells and associated cytokines were elevated in patients with MG and animal models [30,31]. In the present study, we found that the proportion of Th17 cells in the EAMG animal model was significantly higher than that in normal rats, confirming that Th17 cells might also be involved in the pathogenesis of MG.

The physiological functions of these two T lymphocytes are completely different, and maintaining a balanced ratio of these subpopulations is of great importance. The Treg/Th17 balance may play a very important role in the maintenance of immune balance, and damage to this balance may lead to occurrences of autoimmune and inflammatory diseases [32]. Indeed, the presence of Treg/Th17 imbalance has been reported in some autoimmune diseases such as systemic lupus erythematosus and multiple sclerosis [33,34]. In the present study, we detected a decreased percentage of Tregs and an increased percentage of Th17 cells in the EAMG group, indicating that the Treg/Th17 imbalance may also contribute to the pathogenesis of EAMG. Therefore, we speculated that approaches targetted at restoring the Treg/Th17 balance would help to reverse the disease process to some extent, thus achieving a successful therapeutic outcome.

The activated mTOR protein initiates its downstream signaling pathway via a series of complex mechanisms to achieve physiological functions. The functions of the mTOR signaling pathway are extremely complicated, and involve the regulation of numerous biological activities. RAPA is the most common inhibitor targetting mTOR and has been approved for clinical use as a mature drug. Although the mechanism remains unknown, a large number of studies confirmed that RAPA stimulates the proliferation of functional Treg cells [35,36]. In addition, previous studies determined that activation of the mTOR signaling pathway can positively regulate the proliferation and differentiation of Th17 cells [37,38] and that RAPA can prevent Th17 cell proliferation by inhibiting the mTOR pathway. RAPA possibly restores the Treg/Th17 balance by regulating their relative proportions, which consequently inhibits the autoimmune response process to achieve therapeutic purposes.

In the present study, the Treg/Th17 cell imbalance in EAMG was improved by RAPA intervention through increasing the proportion of Treg cells and reducing the proportion of Th17 cells. This suggested that RAPA could achieve a therapeutic effect by correcting the Treg/Th17 imbalance. CTX is a non-specific cytotoxic drug, which exerts its biological effects by interfering with cellular DNA synthesis. Therefore, the proportions of both Treg and Th17 cells in the CTX group decreased. Despite the relief in symptoms, the CTX-treated rats showed a greater weight loss, but higher mortality rate and Lennon scores than the RAPA-treated rats, suggesting that the efficacy of CTX was lower than that of RAPA. However, no significant difference was observed between the groups.

In the present study, the sample size available to calculate peripheral T-cell subsets was small, which might lead to false negative statistical results. Blockage of the mTOR pathway can also inhibit B-cell activation and immunoglobulin production [39] and suppress the proliferation of macrophages and dendritic cells [40]. We speculated that, in addition to regulating cellular immunity, RAPA can inhibit the processes of humoral immunity and antigen presentation, which may also be a mechanism underlying the effectiveness of RAPA treatment for EAMG.

While we did not specifically search for studies regarding MG treatment using RAPA, only one case report study mentioned such therapy. This report described one patient who developed iatrogenic Kaposi’s sarcoma due to the long-term use of azathioprine. The tumor was almost cured and the AChR antibody titer was significantly reduced when the patient switched to RAPA treatment [41]. Compared with traditional immunosuppressive agents, there has been no report on iatrogenic tumorigenesis caused by RAPA. Therefore, RAPA treatment may be more promising and advantageous with respect to long-term prognosis. In addition, previous studies determined that RAPA is effective in the treatment of malignant thymic tumors [42], indicating that RAPA administration might be a better treatment option for patients with MG accompanied by thymoma.

The mTOR signaling pathway has extremely complex functions involved in the regulation of a variety of biological activities, including muscle growth and metabolism [43]. However, in the present study, no significant muscle wasting was detected in rats after RAPA treatment. In addition, there has been no clinical report on RAPA-induced muscle atrophy. Therefore, it can be speculated that the short-term use of RAPA may not cause significant muscle atrophy, although the impact of the long-term use of RAPA on MG prognosis remains to be further determined.

Chauhan et al. [44] found decreased mTOR protein levels in MuSK antibody-positive EAMG muscles and deduced that MuSK protein might be involved in the transcriptional regulation of mTOR protein synthesis. However, whether or not blocking the mTOR signaling pathway further aggravates the muscle atrophy in MuSK antibody positive patients with MG needs to be determined. In addition, whether or not RAPA can be administered to other antibody-positive MG patients remains to be further addressed.

The current study had a few limitations. We only observed the short-term curative effect of RAPA (the observation period was only 10 days). Thus, the Lennon scores were still high after the drug intervention and no rat was cured. We believe that a better curative effect might be detected if the observation period is extended. Future studies are warranted to confirm this hypothesis. This was a preliminary study on utilizing RAPA to treat MG. We are currently focussing on determining post-intervention changes in the RNS, AChR antibody, and muscle and thymus histopathology, which were not assessed in the current study. Thus, we cannot discuss the therapeutic mechanism(s) underlying the effects of RAPA at a deeper level. Future studies will be carried out to identify the mechanism(s) underlying the effects of RAPA on MG in more detail.

Currently, immunosuppressants are widely used for the treatment of MG. As a new immunosuppressant, RAPA has various advantages such as its strong immunosuppressive effect and minimal side effects. In the present study, we demonstrated, for the first time, that RAPA has beneficial effects on an EAMG animal model. Based on these results, we speculate that RAPA would be an effective treatment for MG, but, to our knowledge, no related systematic study has yet been reported.

## Conclusion

The present study confirmed that a Treg/Th17 cell imbalance might be a cause of EAMG. Furthermore, for the first time, we used an mTOR inhibitor, RAPA, to treat AChR antibody positive EAMG rats. We observed symptom improvement in the EAMG animals after treatment, suggesting that the treatment was effective, which might be due to RAPA’s ability to reverse the Treg/Th17 cell imbalance in EAMG. Compared with those treated with CTX, RAPA-treated animals showed a low mortality rate and Lennon scores, but had improved body weights, indicating that RAPA is superior to CTX in treating EAMG to some extent. The mechanism and scope of using RAPA to treat MG requires more in-depth exploration and research. Nonetheless, our study may shed light on MG pathogenesis and treatment.

## Abbreviations

AChR: acetylcholine receptor
CTX: cyclophosphamide
EAMG: experimental autoimmune myasthenia gravis
MG: myasthenia gravis
mTOR: mammalian target of rapamycin
RAPA: rapamycin
RBC: red blood cell
RNS: repetitive nerve stimulation
Treg: regulatory T cell
